# Inducible and Reversible Lentiviral and Recombination Mediated Cassette Exchange (RMCE) Systems for Controlling Gene Expression

**DOI:** 10.1371/journal.pone.0116373

**Published:** 2015-03-13

**Authors:** David C. Bersten, Adrienne E. Sullivan, Dian Li, Veronica Bhakti, Stephen J. Bent, Murray L. Whitelaw

**Affiliations:** 1 School of Molecular and Biomedical Science (Biochemistry), The University of Adelaide, Adelaide, South Australia, Australia; 2 Institute of Molecular Pathology, The University of Adelaide, Adelaide, South Australia, Australia; 3 School of Molecular and Biomedical Science (Genetics), The University of Adelaide, Adelaide, South Australia, Australia; 4 Robinson Research Institute, The University of Adelaide, Adelaide, South Australia, Australia; Max-Delbrück Center for Molecular Medicine (MDC), GERMANY

## Abstract

Manipulation of gene expression to invoke loss of function (LoF) or gain of function (GoF) phenotypes is important for interrogating complex biological questions both *in vitro* and *in vivo*. Doxycycline (Dox)-inducible gene expression systems are commonly used although success is often limited by high background and insufficient sensitivity to Dox. Here we develop broadly applicable platforms for reliable, tightly controlled and reversible Dox-inducible systems for lentiviral mediated generation of cell lines or FLP Recombination-Mediated Cassette Exchange (RMCE) into the Collagen 1a1 (*Col1a1*) locus (FLP-In Col1a1) in mouse embryonic stem cells. We significantly improve the flexibility, usefulness and robustness of the Dox-inducible system by using Tetracycline (Tet) activator (Tet-On) variants which are more sensitive to Dox, have no background activity and are expressed from single Gateway-compatible constructs. We demonstrate the usefulness of these platforms in ectopic gene expression or gene knockdown in multiple cell lines, primary neurons and in FLP-In Col1a1 mouse embryonic stem cells. We also improve the flexibility of RMCE Dox-inducible systems by generating constructs that allow for tissue or cell type-specific Dox-inducible expression and generate a shRNA selection algorithm that can effectively predict potent shRNA sequences able to knockdown gene expression from single integrant constructs. These platforms provide flexible, reliable and broadly applicable inducible expression systems for studying gene function.

## Significance Statement

Inducible expression systems are commonly used to investigate disease relevant gene function. However, significant limitations in current Doxycycline (Dox) inducible ectopic or knockdown gene expression systems often remain, including high background expression in the absence of Dox, low sensitivity to Dox, labour intensive screening of shRNA sequences for knockdown and complex, unreliable establishment of inducible expression systems. We describe simple, modular, and all-inclusive Dox-inducible expression systems delivered through lentiviral infection or Recombination Mediated Cassette Exchange (RMCE), which overcome many previous limitations. We demonstrate improvements in background expression, sensitivity, and shRNA screening, which allow for efficient generation of reliable gain or loss of gene expression systems.

## Introduction

Control of gene expression in mammalian cells and in animal models is of significant importance to basic and applied biological research. The ability to temporally control gene expression in a cell or tissue specific manner enables complex biological questions to be addressed with relevance to disease. While transgenesis or conditional gene knockouts have enabled advances, they are generally limited to permanent and complete gene deletion/ectopic expression and are time consuming, expensive and technically challenging. Doxycycline (Dox)-inducible expression systems have been used widely for ectopic expression and knockdown of genes both in cultured cells and *in vivo*, however significant challenges concerning the reliability of expression in cell lines or animals and significant background expression have limited their use. Recently, site-specific insertion of inducible microRNA-30 context (miR30c) based shRNA cassettes in embryonic stem cells have enabled rapid generation of mice with inducible gene knockdown [1,2]. However, these systems employ Tet activators driven from different genomic sites, do not provide expression in all tissues, and lack a flexible platform to gain tissue-specific expression. Impaired Dox-inducible expression in some tissues may be due to lower promoter activity in these tissues, decreased accessibility of the transcriptional machinery to the loci expressing either the Tet-activator (Tet-On) or the Tet-responsive factor in certain tissues, or limited delivery of Dox to these tissues. In addition, the algorithms used to predict potent miR30c flanked shRNA sequences able to knockdown gene expression from singly integrated constructs are yet to be fully developed, thus time-consuming testing of multiple sequences for each target gene of interest is still required.

Here we generate flexible and reliable Dox-inducible expression systems which can be rapidly employed for GoF and LoF, overcoming difficulties previously encountered with Dox-inducible expression systems. We designed and generated Gateway-compatible lentiviral or Recombination Mediated Cassette Exchange (RMCE) vectors, which contain all the components necessary to gain strong Dox-inducible expression from a near-zero background. We developed a modular platform, which can easily be adjusted to gain tissue-specific expression, and use alternative Tetracycline activators to increase the sensitivity to Dox, which should overcome poor response in some tissues due to limited delivery of Dox. We also designed and tested a miR30c shRNA prediction tool, which enables effective knockdown of target genes from singly integrated Dox-inducible cassettes. These platforms provide a broadly applicable Tet-On system and enable improved methods for rapid generation of cell lines and mice in order to study gain and loss of gene function.

## Material and Methods

### Generation of lentiviral and RMCE constructs

pCMV-Tet-On variant 16 (V16; V9I, F67S, R171K, F86Y, A209T)[3] was kindly provided by A.T. Das, pCMV-Tet-On[4], and Tet-On 3G (V10; F67S, R171K, F86Y, A209T)[3] purchased from Clonetech. Optimised Tet-responsive promoter pTRE3G and pTRE3G-luciferase were also purchased from Clonetech[5]. Tet-On variants were subcloned into pEFIRES-puro [6] using BamHI/EcoRI. pLVTPT plamids were constructed by initially creating pTRE-3G-gtwyA by inserting GtwyA from pEFIRESpuro-eYFP-GtwyA (A gift from S. Furness, Monash University) into pTRE-3G using EcoRV. pTRE-3G-gtwyA-IRES-eGFP was created by digesting GtwyA-IRES-eGFP from pCEMM-NTAP-GtwyA-IRES-eGFP [7] with NheI and inserting it into the NheI site of pTRE-3G. pLV-TRE3G-GtwyA was then created by inserting XhoI/NheI TRE3G-GtwyA into SalI/NheI digested pLV410 (A gift from S. Barry, CHRI, Adelaide) and then MluI PGK-eGFP from pLVx-eGFP-shRNA (A gift from K. Jensen, University of Adelaide) was inserted into MluI digested pLV-TRE3G-GtwyA to create pLV-TRE3G-GtwyA-PGK-eGFP. We then created pLVTPT by inserting EcoRI/NotI Tet-On 3G from pEFIRES-puro-Tet-On 3G into EcoRI/NotI digested pENTR1a, eGFP from pLV-TRE3G-GtwyA-PGK-eGFP was the removed with XhoI/XbaI and replaced with SalI/SpeI Tet-On 3G from pENTR1a-Tet-On 3G. pLV-TRE3G-IRES-eGFP was created by removing TRE3G-GtwyA-IRES-eGFP with XhoI/NheI from pTRE3G-GtwyA-IRES-eGFP and inserted into SalI/NheI digested pLV410. pLVTPTIG was then constructed by first creating pLV-TRE3G-Tet-On 3G-IRES-eGFP by LR recombination between pENTR1a-Tet-On 3G and pLV-TRE3G-IRES-eGFP, then SalI/NheI TRE3G-Tet-On 3G-IRES-eGFP was inserted into pLVTPT using SalI/XhoI to create LVTPTIG. pLVTPTIP was created by digesting pLVTPT with NheI and inserting XhoI digested IRES-Puro PCR product generated from pEF-IRES-puro using Phusion polymerase and EF F 5’GGGTGGAGACTGAAGTTAGGC 3’ IRES-Puro XbaI R 5’TGAGATCTCTAGATCGTGCGCTCCTTTCGGTCG3’.

FLP-Inducer was created by first removing IRES-Puro from pEF-IRES-puro-Tet-On 3G by digestion and religation with EcoRV/BsaBI. Isothermal assembly was then used to insert TRE3G-GtwyA-SV40pA and PGK-ATG-FRT-2xSV40pA PCR products generated from PGK-ATG-FRT (Addgene #20734) and pTRE3G-GtwyA using TRE3G F 5’AAATAGGCGTATCACGAGGCCCTTTCGTCTTCAAGAATTC 3’, TRE3G R 5’GATGTGCGCTCTGCCCACTGACGGGCACCGGAGCCTCACGAAGCTGGCGCGCCTCTAGAGCCGCAGACATGAT 3’, PGK ATG F 5’ctggccttttgctcacatggctcgacagatcccccaattcaCgacctcgaaattctaccg 3’, PGK ATG R 5’ GAATTCTTGAAGACGAAAGGGCCTCGTGATACGCCTATTTagtggtaaactcgatccagac 3’, into HindIII digested pEF-Tet-On 3G. LoxP-STOP-LoxP was generated by nested PCR using the following primers LSL F AflII 5’gatctagtcttaagATAACTTCGTATAGCATACATTATACGAAGTTATgatatctcgatcccccgggtc3’,LSL R XcmI 5’actagatcCCAGTCTAGACATGGATAACTTCGTATAATGTATGCTATACGAAGTTATaagcggccatcgaattcgg 3’, LSL F Iso 5’ttctctccacaggtgtccactcccagttcaattacagctcttaagATAACTTCGTATAGCATACATT3’, LSL R Iso 5’ATTCCAGAGCAGAGTTTATGACTTTGCTCTTGTCCAGTCTAGACATGGATAACTTCG 3’ and inserted into AflII/XcmI FLP-Inducer plasmids by isothermal assembly. Human Synapsin I promoter (hSynI) was amplified from pLenti-Synapsin-hChR2(H134R)-EYFP-WPRE (Addgene #20945) using hSyn Iso F 5’AACTCATCAATGTATCTTATCATGTCTGCGGCTCTAGAGGtaagtgtctagactgcagagggc 3’ and hSyn Iso R 5’ gctagcctatagtgagtcgtattaagtactctagccttaagtttctcgactgcgctctcagg 3’, and human Glial Fibrillary Acidic Protein (GFAP) promoter was amplified from human genomic DNA using GFAP Iso F 5’ AACTCATCAATGTATCTTATCATGTCTGCGGCTCTAGAGGcccacctccctctctgtgc 3’, and GFAP Iso R 5’GCTAGCCTATAGTGAGTCGTATTAAGTACTCTAGCCTTAACCTGCTCTGGCTCTGCTCG 3’and inserted into AscI/AflII digested FLP-Inducer by isothermal assembly. Finally the CAG promoter was inserted into FLP-Inducer by first removing CAG from pCAG-ASIP using XhoI/BamHI and inserting it into SalI/BamHI digested pEYFP-ACN (Addgene #20344). We then removed the CAG promoter using AscI/AflII and ligated it into AscI/AflII digested pFLP-Inducer.

pENTR1a-dsRed-m30c was constructed by first cloning dsRed-Express (Clonetech) into pENTR1a (Invitrogen) with SalI/NotI, then the miR30 based context was cloned into NotI/XbaI sites of pENTR1a-dsRed using the following primers: miR30 5’Arm 5’cgtaaGCGGCCGCGTCGACTAGGGATAACAGGGTAATTGTTTGAATGAGGCTTCAGTACTTTACAGAATCGTTGCCTGCACATCTTGGAAACACTTGCTGGG 3, miR30 mid Arm 5’CTTGGAAACACTTGCTGGGATTACTTCTTCAGGTTAACCCAACAGAAGGCTCGAGCAACCAGATATCGAATTCAAGGGGCTACTTTAGGAGCAATTATCTTGTTTACT 3’, miR30 3’Arm 5’GGAGCAATTATCTTGTTTACTAAAACTGAATACCTTGCTATCTCTTTGATACATTTTTACAAAGCTGAATTAAAATGGTATAAATTAAATCACTTTCTAGAcgtaa 3’. pENTR1a-eYFP-m30c and pENTR1a-dnucTomato-m30c were subsequently constructed by replacing the dsRed with KpnI/NotI eYFP or SmaI/NotI dnucTomato from peYFP-ACN and pNESN (A gift from A. Yoo and G.R. Crabtree, Stanford University), respectively.

miR30 shRNA sequences were then inserted into pENTR1a-dsRed-m30c or pENTR1a-mnucTomato-m30c by digesting the plasmid with XhoI/EcoRV and inserting PCR generated ~190bp products using 97mer template shRNA primers (Ultramer IDTdna, S1 Table) and M30c Iso F 5’CTTGCTGGGATTACTTCTTCAGGTTAACCCAACAGAAGGCTCGAGaaggtatatTGCTGTTGACAGTGAGCG3’ and M30c Iso R 5’AACAAGATAATTGCTCCTAAAGTAGCCCCTTGAATTCGATTCCGAGGCAGTAGGCA3’ amplification primers using isothermal assembly as described [8]. Multiplex cloning of 10 or more shRNA clones was achieved by mixing 190bp generated shRNA cloning templates and assembly into XhoI/EcoRV digested pENTR1a-miR30c plasmids. Individual shRNA clones were then identified by sequencing. Using this technique cloning efficiency was close to 100% and 50–70% of these could be confirmed by sequencing. Fluorescent miR30-shRNA or Flag tagged NPAS4, SIM2s and SIM2l cDNAs were recombined into pFLP-Inducer or pLVTPT vectors by LR recombination.

### Lentiviral production, infections and cell line generation

Lentivirus was produced by transient transfection in human embryonic kidney (HEK) 293T cells using calcium phosphate precipitation, and for primary neuron infections was concentrated (0.5–1x10^9^ TU/ml) and viral titre estimated as described [9]. Stable cell lines were produced by infecting cells with virus plus 8 Stapolybrene at <20% infection rate and subsequently selected in Puromycin (200–1000 ng/ml). Primary mouse embryonic day 16 cortical neurons were infected with for overnight and washed 3 times in Neurobasal/ 2% B27/1% Penicillin-Streptomycin/1% Glutamine media.

### Cell culture, Doxycycline inductions and transfections

HEK293T and MEFs (mouse embryonic fibroblasts) were cultured in DMEM (Gibco #12430), 10%FBS, 1% GlutaMAX (Gibco), 1% Penicillin/Streptomycin (Invitrogen), while LNCaP, DU145, MDA-MB-231, MDA-MB-453, MCF7 cells were cultured in RPMI 1640 (Gibco #11835) with the same additives. Mouse embryonic stem (mES) cells were maintained on irradiated primary MEFs in DMEM (Gibco #11995), 15%FBS, 1% L-Glutamine, 1% Penicillin/Streptomycin, 55μM β-Mecaptoethanol (Gibco #21985) and 1:10,000 ESGRO (LIF; Millipore). Transfections were carried out according to manufacturer instructions using Fugene6 (Roche) unless otherwise indicated. Expression in cell lines and reporter experiments used induction with 1μg/ml Doxycyline (Sigma) unless otherwise indicated. Immortalised *Maged1* knockout MEFs were a kind gift from Prof. Olivier de Backer, University of Namur, Belgium.

### Luciferase reporter gene assays

~1x10^5^ HEK293T cells were transfected with 50ng of pEF-IRES-puro-Tet-On/Tet-On V16/Tet-On 3G, 200pg pCMV-RL and 200ng of pTRE3G-Luc with Fugene6 (Roche). Cells were lysed 48hrs after transfection and firefly and renilla luciferase were measured on a GloMax96 Microplate Luminometer using the Dual Luciferase Assay System (Promega).

### Col1a1 targeting and screening of mouse embryonic stem cells

C57B6/Sv129 hybrid mouse embryonic stem cells were used to target the Col1a1 locus with pCol1A-frt-hygro-pA (Addgene # 20730) by homologous recombination essentially as described [10,11]. Embryonic stem cell genomic DNA was digested with EcoRI and an XcmI fragment from pCol1A-frt-hygro-pA was used as a southern probe to identify correctly targeted colonies, which were termed *mES-Col1a1-FLP-In* cells.

### Recombination mediated cassette exchange (RMCE) in mES-Col1a1-FLP-In cells

0.8–1.5 x 10^7^ mES-Col1a1-FLP-In cells grown on MEFs were electroporated twice using a Biorad x-cell gene pulse electroporator set at 400V and 125 μF with 25μg of pPGK-FLPo-bpA (Addgene #13793) and 50μg of pFLP-Inducer plasmid and plated on irradiated Hygromycin resistant MEFs (Millipore). 36–48hrs post electroporation cells were incubated with 140 μg/ml Hygromycin containing media and colonies picked typically after 6–8 days of selection. Using this technique we were able to generate ~30 Hygromycin resistant colonies per electroporation, all of which were able to induce expression in the presence of Dox.

### CRE mediated excision of LoxP-STOP-LoxP from mES-FLP-Inducer-LSL cells

1 x 10^7^ mES-FLP-Inducer-LSL-dsRed-m30c-shP53(814) cells were electroporated using a Biorad x-cell gene pulse electroporator set at 250V and 500 μF with 40μg of pCAG-GS-Cre plasmid. 2 days after electroporation the cells were treated with 2 μg/ml Dox for 24hrs or left untreated and cells were imaged using fluorescence microscopy. Genomic DNA was extracted before and after electroporation with the CRE expressing plasmid for genotyping.

### Cell fluorescence and FACS analysis

Cell fluorescence was visualised using an inverted microscope (Zeiss) and images manipulated and overlayed using ImageJ software. FACS analysis was performed on a BD Canto flow cytometer and data analysed using FlowJo software.

### RNA extraction, cDNA synthesis, and Real-time PCR

Cells were lysed in Trizol (Invitrogen) and RNA was isolated, purified and reverse transcribed using superscript III (Invitrogen) and cDNA was diluted for real time PCR. Real-time PCR was performed in triplicate using primers specific to *Bdnf* exon I F 5’ TTGAAGCTTTGCGGATATTGCG 3’, *BDNF* exon I R 5’ AAGTTGCCTTGTCCGTGGAC 3’ *Npas4[12]*, *hSIM2L* F 5' AGCAGCTCGTCTCCAGCTAA 3', *hSIM2L* R 5' GTGTCCTCGCCGAACCTG 3', h*SIM2s* [13], human RNA polymerase 2A (*POLR2A*)[14] or *GAPDH[12]* and spanned an intron where possible. The expression level was normalised to RNA polymerase 2A and presented as fold induction over parent cell line. Melt curves of PCR products were used to confirm a single amplicon.

### Western Blot

Lysates were prepared in 20mM HEPES pH 8.0, 420mM NaCl, 0.5% Igepal, 25% Glycerol, 0.2mM EDTA, 1.5mM MgCl_2_, 1mM DTT, and protease inhibitors, separated on 7.5–10% SDS-PAGE gel and transferred to nitrocellulose. Proteins were detected using anti-FLAG (Sigma, M2), anti-ARNT1 (Abcam, ab2), anti-P53 (Calbiochem, Ab-1) anti-ARNT2 (Santa Cruz, M-165) and anti-α-Tubulin antibodies (Serotec, MCA78G). Primary antibodies were detected using horseradish peroxidise-conjugated secondary antibodies and visualised using chemiluminescence.

### shRNA design algorithm

Our approach for predicting the efficacy of candidate shRNAs was based on an implementation of a support vector machine (SVM) using the statistical programming language R and the e1071 module. The bases of the candidate siRNA guide strand sequences were encoded using the method described by Sciabola *et al*, 2012 [15], and flanking regions corresponding to 10 bases on the 5’ end of the guide sequence (3’ in the mRNA sequence) and 16 bases on the 3’ end of the guide sequence (5’ in the mRNA sequence) were included in the SVM data table. Additional data incorporated into the data sets included the siRNA scoring output from mir-scan [16], the posterior probability output from contrafold [17], and the output from sfold’s sirna module[18]. The source code for the shR-Thing algorithm is available at https://code.google.com/p/shr-thing/

### Generation of a miR30 shRNA selection algorithm

The training data set comprised the reported knockdown efficacy data sets from Fellmann *et al*, 2011 [19] and Tan *et al*, 2012 [20], using the ProdEn score reported by Fellmann, and a transformed score with similar properties to Fellmann’s ProdEn score, generated by averaging the log2-transformed hybridization and sequencing values from the sort 6 round, taking 2 to the power of that (removing the log transformation), multiplying by 4, and subtracting 10. This process was empirically determined to yield a similar distribution of scores from the Tan data set as Fellmann’s ProdEn score.

The SVM was trained using the Fellmann and Tan data sets using eps regression and a radial kernel. The SVM was tuned, but shown not to be greatly sensitive to the cost and gamma parameters, which were set at 2.25 and 0.1, respectively. The oligo sequences that received the highest scores from the SVM run were checked manually to exclude homopolymers greater than length 4, and the hairpin sequence was generated following Dow *et al*, 2012 [1].

### Statistical analysis

Data was graphed and statistical analysis performed using GraphPad Prism 5 (GraphPad Software Inc.). Statistical differences were evaluated by the Student’s t-test with the level of significance set at p <0.05.

### Ethics

To generate primary mouse neuron cultures, pregnant mice were CO_2_ euthanatized and embryonic cortices were dissected with approval from the University of Adelaide animal ethics committee (Ethics approval # 0000009506).

## Results

### Reduced background expression and improved sensitivity to Doxycycline using the 3^rd^ generation Tet-activator, Tet-On 3G

**Fig 1:**
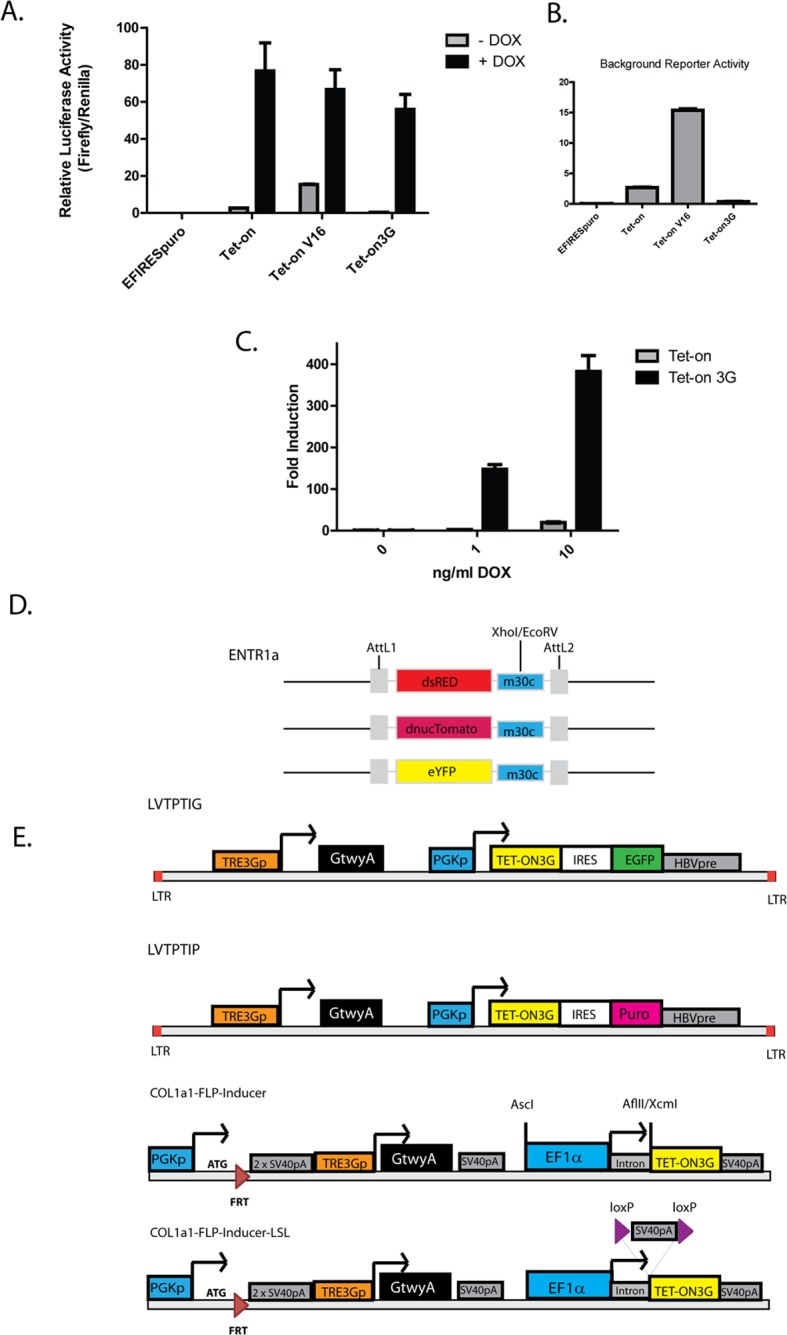
Construction of sensitive, low background Dox-inducible lentiviral and Flp-In systems. (a) Tetracycline-responsive luciferase reporter gene assays in HEK293T cells using Tet-On variants with (black) or without (grey) 1μg/ml Doxycycline for 24hrs. (b) Expanded background luciferase activities in the absence of Doxycycline from (a). Data are mean relative luciferase activities ± SEM of 3 independent experiments. (c) The sensitivity of Tet-On or Tet-On 3G to Doxycycline using a Tetracycline-responsive luciferase reporter gene in HEK293T cells in the presence (black) or absence (grey) of indicated concentrations of Doxycycline. Data show fold inductions of mean relative luciferase for Doxycycline treated versus non-treated cells, ± SEM of 3 independent experiments. (d) Schematic of vectors for fluorescent miR30c donor plasmids containing dsRed, dnucTomato, or eYFP. (e) Schematic of vectors for Lentiviral (LVTPTIG and LVTPTIP) and FLP-Inducer (Col1a1-FLP-Inducer and Col1a1-FLP-Inducer-LSL) Doxycycline-inducible destination plasmids.

Significant improvements have recently been made to the Tet-On inducible expression systems, including the molecular evolution of high sensitivity, low background variants of the Tet-activator and low background Tet- responsive promoters [3,5]. Before incorporating these components into lentiviral or RMCE expression systems, we confirmed these improvements to the Tet-activator using luciferase reporter assays containing a minimal CMV promoter and seven optimised Tet response element (TRE3G) repeats [5]. We observed strong Dox-inducible expression from all Tet-On variants (Fig. 1a), however significant background “leaky” expression was observed in the absence of Dox when either the wildtype (WT) Tet-On or the Tet-On V16 variants were employed as Tet-activators in HEK293T cells (Fig. 1b). Activation of the TRE promoter in the absence of Dox was negligible when the Tet-On 3G variant was used (Fig. 1b). The inability to gain Dox-inducible expression in some tissues, as described by several groups using the Tet-activator system, has been suggested to be due to low Dox concentration in these tissues. This issue may be overcome by using more sensitive Tet-activator variants [2,21,22]. We therefore tested the sensitivity of Tet-On 3G to Dox, and observed that the Tet-On 3G variant was active at Dox concentrations as low as 1ng/ml, revealing dramatically increased sensitivity to Dox compared to the Tet-On (Fig. 1c).

### A flexible lentiviral platform for Dox-inducible shRNA and cDNA expression in primary and immortalised cells

Next we sought to incorporate the optimised Tet-On 3G variant and TRE3G promoter into flexible platforms that would enable inducible expression or knockdown in readily generated stable cell lines or transduced primary and post-mitotic cells. We initially observed that generation of stable cell lines using a two plasmid system, where the Tet-activator and the TRE promoter-driven response gene were delivered on separate plasmids, was not only time consuming but resulted in cell lines displaying heterogeneous Dox-inducibility (data not shown). We reasoned that creation of constructs where all the components were present in *cis* would expedite cell line generation and allow homogenous inducibility in all cells. We placed the TRE3G promoter upstream of a Gateway A exchange cassette, followed by the constitutive Phosphoglycerate Kinase-1 promoter (PGKp) to drive expression of the Tet-On 3G and either the EGFP or Puromycin resistance gene linked by an internal ribosome entry site (IRES) (termed LVTPTIG and LVTPTIP, respectively; Fig. 1e). To allow for broad applications, such as use in primary or post-mitotic cell types and rapid generation of stable cell lines, which may prove refractory to transfection, we utilised a third-generation lentiviral platform to deliver the expression cassette (Fig. 1e). We also generated several Gateway donor plasmids containing dsRED, nucTomato, or EYFP with a downstream miR30c cassette, for producing shRNA sequences that are formatted for processing to siRNA to achieve gene knockdown (Fig. 1d).

**Fig 2:**
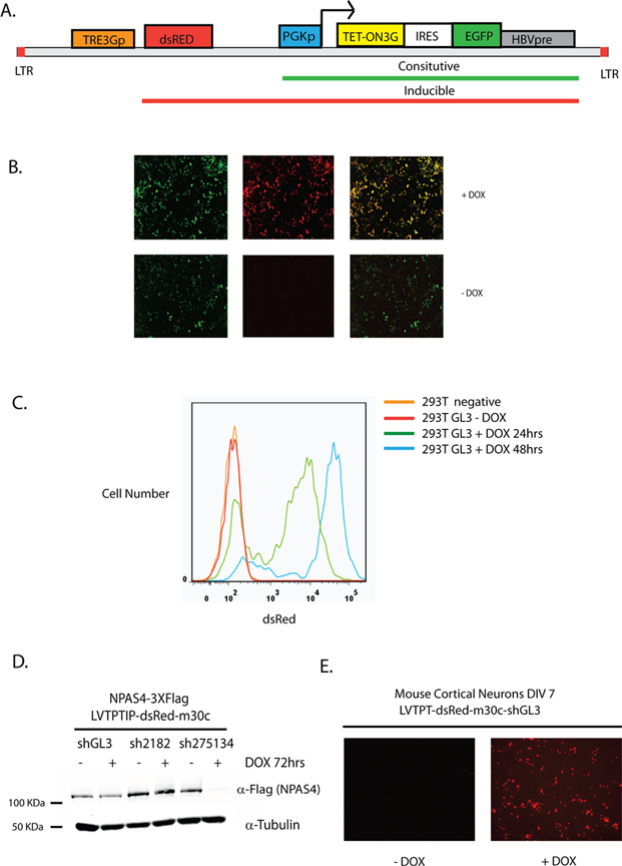
Lentiviral LVTPT system allows for inducible cDNA and shRNA expression in stable cell lines and primary neurons. (a) Vector diagram of constitutively expressed (Tet-On 3G and eGFP) and Doxycycline-inducible (dsRed (cDNA), Tet-On 3G and eGFP) components of the LVTPT system (b) dsRed (left), eGFP (middle), or merged (right) fluorescence images of HEK293T cells infected with LVTPTIG-dsRed virus in the absence (lower panel) or presence (upper panel) of 1μg/ml Doxycycline for 24hrs (c) FACS analysis of dsRed expressing cells in uninfected (orange) or HEK293T cells stably infected with LVTPTIP-dsRed-shGL3 and selected with Puromycin in the absence (red) or presence of 1μg/ml Doxycycline for 24 (green) or 48 (blue) hrs. (d) LVTPTIP elicits effective RNAi in HEK293T cells. HEK293T cells stably infected with LVTPTIP-dsRED-shGL3 (negative control), LVTPTIP-dsRED-shNPAS4 2182 (impotent shRNA), or LVTPTIP-dsRED-shNPAS4 275134 (potent shRNA) were transfected with expression vector pEFBOS-NPAS4–3xFlag and treated with (+) or without (-) 1 μg/ml Doxycycline for 72 hrs. Whole cell extracts were analysed for NPAS4 (anti-Flag) and tubulin levels by immunoblot. (e) dsRed fluorescence images of embryonic day 16 mouse cortical neurons cultured *in vitro* and infected with concentrated LVTPT-dsRed-shGL3 virus and treated with (right panel) or without (left panel) 1μg/ml Doxycycline for 24hrs.

**Fig 3:**
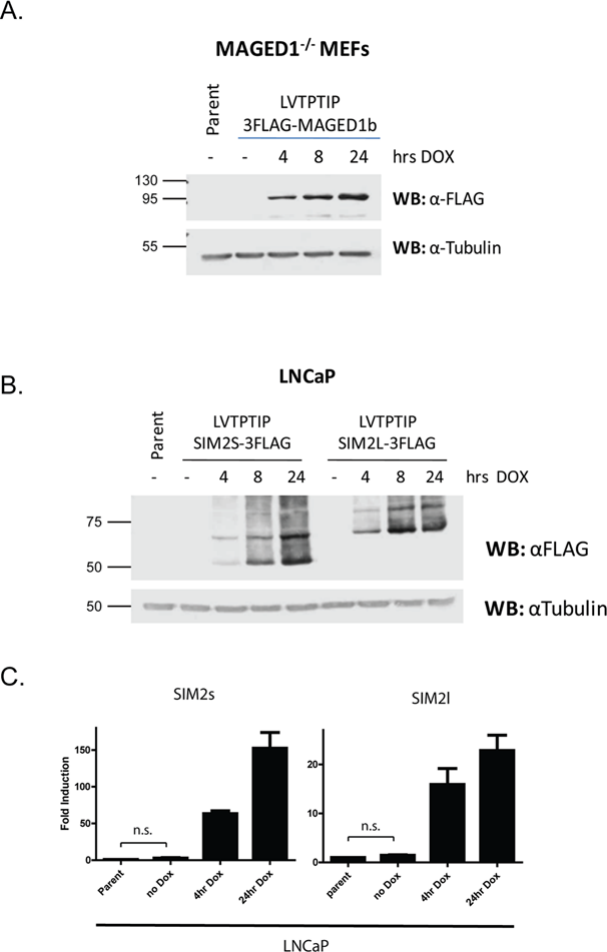
Efficient generation of Doxycyline-inducible stable cell lines free of background expression. (a) Knock out *Maged1* Mouse Embryonic Fibroblasts (MEFs) were stably transduced with LVTPTIP-3xFlag-MAGED1b and treated with 1μg/ml Doxycycline for 4, 8 or 24hrs. Whole cell extracts were analysed for MAGED1b (anti-Flag) and tubulin by immunoblot. (b) LNCaP prostate cancer cells were stably transduced with LVTPTIP-SIM2s-3xFlag or LVTPTIP-SIM2l-3xFlag and treated with 1μg/ml Doxycycline for 4, 8 or 24hrs. Whole cell extracts were analysed for SIM2s or SIM2l (anti-Flag) and tubulin by immunoblot. (c) mRNA of SIM2s and SIM2l from LNCaP prostate cancer cells from (b) was measured by Real-time PCR and normalised to RNA polymerase subunit 2A (POLR2A). Data are mean normalised mRNA fold induction over parent cell line ± SEM of 3 independent experiments. Statistical significance was determined using an unpaired student t-test. n.s. (not significant).

These lentiviral constructs were efficient in producing high virus titre and transducing a number of different mouse and human cell lines (Figs. 2, 3 and S1 Fig.). Initially, we tested these constructs in HEK293T cells stably transduced with a construct containing dsRed downstream of the TRE3G promoter (Fig. 2a). In the absence of Dox, dsRed was undetectable, while EGFP driven from the PGK promoter was expressed in transduced cells (Fig. 2b). After 24hrs of Dox treatment dsRed was robustly expressed throughout the EGFP-expressing population, indicating that the lentiviral constructs can produce complete populations of expressing cells (Fig. 2b). As previously reported, the Dox-induced expression also increased expression from the downstream PGK promoter as apparent by an increase in EGFP signal (Fig. 2b). This is presumably due to transcriptional read-through from the binding and activation of Tet-activator and the lack of polyadenylation sequences in lentivector constructs [23,24] (Fig. 2b). This allows for positive feed-forward expression of the Tet-On 3G, strengthening induction from the Tet-responsive TRE3G promoter.

To investigate the inducible expression of these constructs further, we used the LVTPTIP lentivector to generate stable HEK293T cell lines harbouring the cDNA for dsRed fused with miR30c shRNA cassettes targeting either luciferase (shGL3) or the basic Helix Loop Helix /Per-Arnt-Sim homology domain (bHLH/PAS) transcription factor neuronal PAS domain protein 4 (*Npas4)* (sh2182 and sh275134). We transduced the HEK293T cells such that only 20–30% of cells were infected, limiting the number of integrants per cell to approximately 1 [25]. The inducibility of the dsRed-shGL3 cell line was assessed using FACS analysis. In the absence of Dox, dsRed fluorescence was completely absent from all cells and indistinguishable from parental HEK293T cells (Fig. 2c). After 24hrs of Dox treatment most of the cells became RFP positive, and by 48hrs the vast majority of cells strongly expressed RFP (Fig. 2c). To assess whether Dox-inducible expression from constructs integrated at low copy number in cell lines was sufficient to knockdown gene expression, we transiently overexpressed Flag-tagged NPAS4 in stable HEK293T cell lines and induced shRNA expression for 72hrs with Dox (Fig. 2d). We observed potent knockdown of NPAS4 in sh275143-expressing cells (high effect siRNA) but not in control shGL3 or sh2182-expressing cells (low effect siRNA) (Fig. 2d). We were also able to generate very high titre (0.5–1x10^9^ TU/ml) using this lentivector platform, which enabled its use in primary cell transduction experiments. We cultured primary mouse embryonic day 16 post mitotic cortical neurons *in vitro* and successfully transduced them with LVTPT-dsRed-shGL3 lentivirus, gaining strong Dox-inducible expression in the vast majority of neurons (Fig. 2e). This suggests our platform will be useful for primary cell or *in vivo* inducible LoF and GoF experiments.

In addition to achieving successful shRNA production, we generated several different cell lines bearing inducible cDNA expression. Robust, inducible expression has been observed in 8 different cell types with a range of cDNAs. For example, Melanoma Antigen Family D isoform 1b (MAGED1b) was reintroduced into *Maged1* knockout mouse embryonic fibroblasts (MEFs) using LVTPTIP lentivector transduction and Puromycin selection. We observed strong expression within 4hrs of Dox treatment from a background free of detectable expression in the absence of Dox (Fig. 3a). In addition, the LVTPTIP lentivector provided inducible expression of both the short and long isoforms of the bHLH/PAS transcription factor Single Minded 2 (*SIM2s* or *SIM2l*) in prostate and breast cancer cell lines (Fig. 3b and S1 Fig.). These lines consistently showed strong Dox-inducible expression within 4 hrs of treatment with minimal or no background expression in the absence of Dox (Fig. 3b and S1 Fig.). The lack of any detectable expression in the absence of Dox is of particular importance when working with genes detrimental to cell survival or linked to growth and differentiation. We therefore analysed the expression of *SIM2* mRNA by quantitative real time PCR in stable LNCaP prostate cancer cell lines to determine whether there was any background expression that was undetectable by western blot. Importantly, we observed no significant (p = 0.18 *SIM2s*, p = 0.35 *SIM2l*) background expression in the absence of Dox when compared to parental cell lines (Fig. 3c), indicating that in general these platforms could be used to investigate ectopic expression of genes which function in apoptosis, or shRNA production which targets genes essential for cell survival, growth or differentiation, for example members of the bHLH/PAS transcription factor family [26].

### Generation of FLP-In *Col1a1* targeted mouse embryonic stem cells

**Fig 4:**
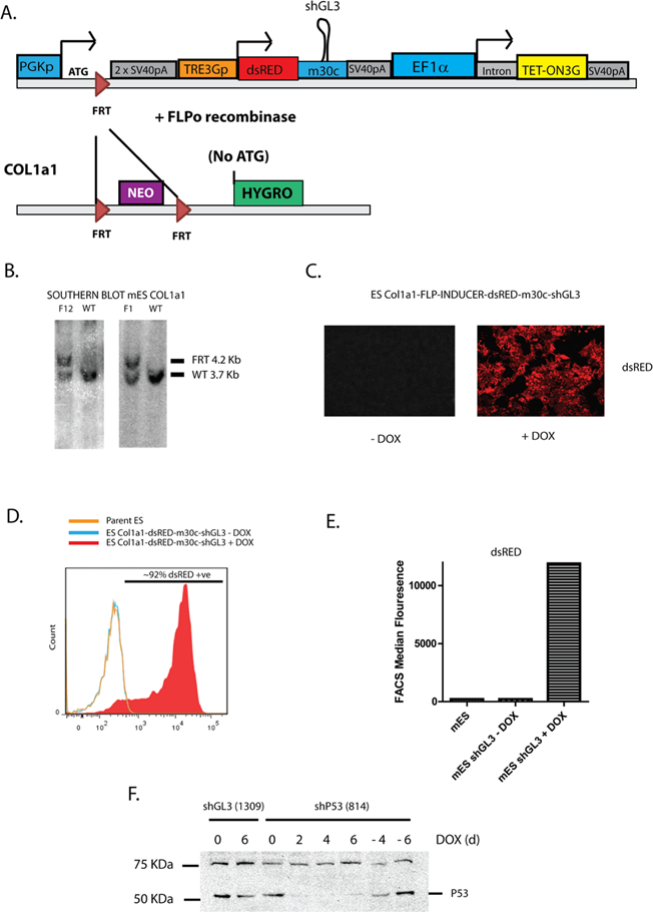
Inducible and reversible expression and knockdown from the Col1a1 locus using the FLP-Inducer system. (a) Schematic representation of Col1a1-FLP-In targeting strategy. Electroporation of pFLP-Inducer-dsRed-shGL3 and pFLPo recombinase into mouse embryonic stem (mES) cells pre-targeted with Col1a1 FRT Hygromycin (–ATG) (promoter) enables gain of Hygromycin resistance and site specific integration of the FLP-Inducer construct. (b) Southern Blot of two mouse embryonic stem cell lines containing Col1a1-PGK-FRT-neo-FRT (–ATG) Hygro targeted to the *Col1a1* locus and two G418 resistant WT lines, using a 5’ internal probe. (c) dsRed microscopy of a Col1a1-FLP-Inducer- dsRed-shGL3 Hygromycin-resistant mES cell line in the presence (right panel) or absence (left panel) of 1μg/ml Doxycycline for 24hrs. (d) FACS analysis of parent mES Col1a1 FRT Hygromycin (–ATG) (promoterless) cells (Orange) or mES Col1a1-FLP-Inducer- dsRed-shGL3 cells (blue and red) in the presence (red) or absence (blue and orange) of 1μg/ml Doxycycline for 24hrs. (e) Median dsRed fluorescence in FACS analysis in (d). (f) shRNA mediated knockdown of P53 from the Col1a1-FLP-Inducer mES cells. Col1a1-FLP-Inducer-dsRed-shGL3 or Col1a1-FLP-Inducer-dsRed-shP53 were incorporated into the Col1a1 locus in mES cells by recombination mediated cassette exchange (RMCE) and shRNA induced using 1μg/ml Doxycycline, then allowed to recover by Doxycycline removal for indicated periods. P53 and tubulin protein expression were determined by immunoblot.

In addition to lentiviral-mediated gene delivery to primary neurons and generation of stable cell lines, we were also interested in applying this technology to generate inducible expression from a defined locus in mouse embryonic stem (mES) cells. These modified mES cells can be used to study function of genes during *in vitro* differentiation protocols and in generating mouse models with inducible gain and loss of gene function. Following methods employed by the Jaenisch lab [11] we used a region 3’ of the coding sequence in the *Col1a1* locus for targeted homologous recombination to insert a FLP/*FRT* based RMCE (Recombination Mediated Cassette Exchange) construct into hybrid S129/C57BL6 mES cells. RMCE allows rapid and site-specific exchange of single Dox-inducible cassettes into the *Col1a1* locus and has been used to generate transgenic animals with ubiquitous Dox-inducible expression [11,27]. The *Col1a1* targeting construct contained a Neomycin selection cassette flanked by *FRT* sites, followed by a Hygromycin cassette lacking a promoter or start codon as used by Jaenisch and colleagues [11,27] (Fig. 4a). We generated two independently derived mES cell lines by homologous recombination (F1 and F12 mES cell lines) and screened for correctly targeted single integrants by southern blot (Fig. 4a and 4b).

### Inducible and reversible shRNA and cDNA expression from the *Col1a1* locus using an optimised Flp-In/Tet-Activator system: pFLP-Inducer

Next we generated a platform to facilitate highly efficient FLP-mediated insertion of Dox-inducible cassettes into the *Col1a1* locus from a single construct. These constructs contain a PGK promoter followed by an ATG translation start codon, a lone FRT site and two SV40 polyadenylation (PolyA) sequences to stop transcription from the endogenous *Col1a1* gene (Figs. 1e and 4a). Downstream of this is the TRE3G promoter, Gateway A cloning cassette, an SV40 PolyA sequence and a strong constitutive promoter (Human elongation factor-1 alpha [EF1α] or CMV early enhancer/chicken beta actin [CAG]) driving the expression of the Tet-On 3G activator followed by an SV40 polyA sequence. We engineered specific AscI and AflII restriction sites to flank the promoter driving the Tet-On 3G to allow for efficient promoter exchange, and an XcmI site before the Tet-On 3G start codon to allow insertion of a LoxP-flanked SV40 PolyA (LSL) sequence to stop transcription (Fig. 1e). We have created a number of different Gateway-compatible Flp-In cassettes with either strong constitutive promoters followed by LSL (EF1α and CAG) or tissue-specific promoters (human Glial Fibrillary Acidic Protein [hGFAP], astrocytes; human Synapsin I [hSynI], neurons; modified minimal rat Probasin promoter [ARR2Pb], prostate), to allow for use in tissue/cell-specific Dox-inducible applications (Fig. 1e, S2 and S3 Figs.). Electroporation of the Flp-In construct along with the codon optimised FLP (FLPo)-expressing vector [28] generally resulted in highly efficient (near 100%) generation of approximately 30 Hygromycin-resistant, Dox-inducible colonies when using RMCE S129/C57BL6 cells.

We have generated more than 30 separate dsRed miR30c shRNA-expressing mES lines using the Flp-Inducer constructs and consistently observed strong Dox-inducible expression in all Hygromycin-resistant lines generated (Fig. 4c and 4d). Using FACS analysis and fluorescence microscopy we showed that background fluorescence was indistinguishable from that of the parental mES RMCE line and induction of dsRed expression was largely uniform and reversible (Fig. 4c, 4d, 4e; and S4 Fig.). We also tested the ability of these FLP-Inducer mES cell lines to efficiently knockdown gene expression when producing shRNA from a single site in mES cells by using a well-defined potent shRNA against p53(814)[19]. Within 2 days of Dox addition we observed an almost complete knockdown of p53 protein, which was not observed using a non-specific shRNA (shGL3). Knockdown of p53 protein was maintained in the presence of Dox for 6 days and reversed after 4–6 days of Dox withdrawal (Fig. 4f), demonstrating the efficacy and reversibility of this system.

### Optimisation of shRNA prediction for loss of gene function experiments

We noted that the success of LoF experiments using this system largely relies on the use of potent and specific miR30c shRNAs. shRNAs produced ectopically from a single copy expression cassette that achieve high knockdown efficiency are rare, and as yet such sequences are difficult to reliably predict using current siRNA design algorithms. Recently, a number of high throughput experiments have been performed to identify potent shRNA sequences which, when embedded with the miR30-based context, successfully produce functional siRNAs [19,20]. We utilised this data, which assesses the potency of ~60,000 miR30c shRNA sequences, and added variables to account for entropy and mRNA fold data in order to develop an updated siRNA selection algorithm to identify potent miR30c shRNA sequences.

**Fig 5:**
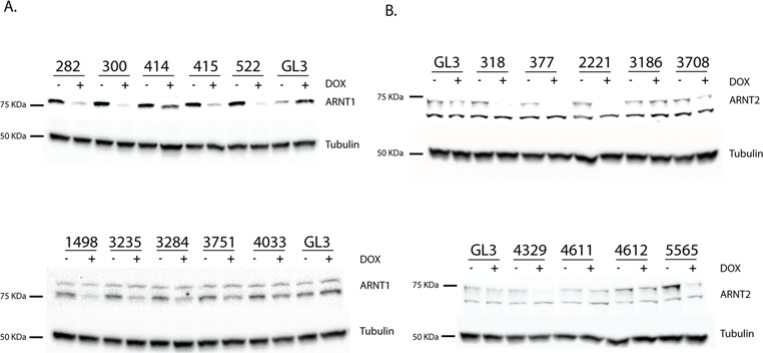
Efficient prediction of shRNA sequences for knockdown of ARNT1 or ARNT2 in mES_FLP-Inducer cells. Induced shRNA knockdown of (a) ARNT1 or (b) ARNT2 in mES-FLP-Inducer-nucTomato-m30c-shRNA cells treated with 1μg/ml Dox for 48hrs (+) versus untreated (-). Western blotting was used to detect ARNT1, ARNT2, and tubulin using α-ARNT1, α-ARNT2, or α-tubulin antibodies, respectively.

We tested the ability of this shRNA selection algorithm to effectively predict efficient knockdown from singly integrated constructs using our mES FLP-Inducer system. We choose to target two genes, *Arnt* and *Arnt2*, which express bHLH/PAS transcription factors in embryonic stem cells [29] and can be readily detected by western immunoblot. We cloned the top 10 shRNA sequences predicted by our algorithm against either *Arnt* or *Arnt2* using a multiplex cloning technique (see methods) and generated mES-FLP-Inducer cell lines for each construct. We then tested each shRNA for knockdown of ARNT or ARNT2 protein following 48 hrs of Dox treatment and observed ~60% (12/20) of shRNA constructs could effectively knockdown gene expression (Fig. 5a and 5b).

**Fig 6:**
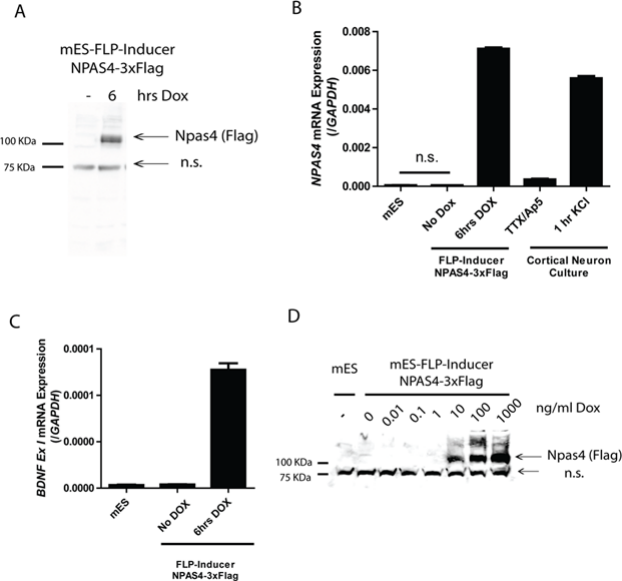
Inducible, background free expression of cDNA from the *Col1a1* locus (a) Col1a1-FLP-Inducer-mNPAS4–3xFlag mouse embryonic stem (mES) cells were cultured in the absence or presence of 1μg/ml Dox for 6 hrs and whole cell extracts analysed for NPAS4–3xFlag by immunoblotting with anti-Flag antibodies. n.s. represents a nonspecific band (b) *Npas4* mRNA was measured using real time PCR of Col1a1-FLP parental cells, Col1a1-FLP-Inducer-mNPAS4–3xFlag cultured in the absence or presence of 1μg/ml Dox for 6hrs, and mouse embryonic day 16 (E16) cortical neurons cultured *in vitro* and pre-treated with 2μM Tetrodotoxin (TTX) and 100μM D (-)-2-amino-5-phosphopentanoic acid (AP-5) (TTX/AP5) channel blockers for 24hrs prior to 55mM KCl depolarisation treatment for 1hr. Data are mean ± SD of 3 independent experiments. Statistical analysis was calculated using two-tailed students t-test of 3 independent experiments. not significant (n.s.) (c) *Bdnf* mRNA was measured using real time PCR of Col1a1-FLP parental cells and Col1a1-FLP-Inducer-mNPAS4–3xFlag cultured in the absence or presence of 1μg/ml Dox for 6hrs (d) Col1a1-FLP parental cells (mES) or Col1a1-FLP-Inducer-mNPAS4–3xFlag cells were cultured in the absence or presence of indicated concentrations of Dox for 24 hrs and whole cell extracts analysed for NPAS4–3xFlag by immunoblotting with anti-Flag antibodies. n.s. represents a nonspecific band.

Inducible expression of tagged proteins is of particular use in studying transcription factor occupancy of target sites in the genome and transcription factor function in protein complexes. However, excessive overexpression and issues with temporal control or background expression can lead to false positive discovery in such experiments. We therefore created FLP-Inducer cell lines containing Flag-tagged *Npas4* to assess background and inducible expression levels of a prototypical cDNA in this system. We observed rapid induction of NPAS4–3xFlag protein expression following 6hrs of Dox addition in the mES cells (Fig. 6a). *Npas4* mRNA and protein expression is highly restricted to the brain and upregulated following neuronal depolarisation [30,31]. To assess the level of expression compared to endogenously expressed *Npas4* we compared mRNA of *Col1a1*-FLP-Inducer mES cells to mouse cortical neurons cultured *in vitro* and depolarised with 55mM KCl for 1hr. After 6hrs of Dox treatment *Npas4* mRNA levels were similar to the endogenous levels in KCl depolarised neurons (Fig. 6b). Importantly, we also observed no statistical difference between expression of *Npas4* in parental mES cells or untreated mES-FLP Inducer-NPAS4–3xFlag cells (Fig. 6b) indicating a distinct lack of background “leaky” expression compared to previous Tet-On systems expressing cDNAs from the *Col1a1* locus [32,33]. Ectopic NPAS4 expression in mES-FLP-Inducer–NPAS4–3xFlag cells was also able to activate the downstream target gene *Bdnf* after 6hrs of Dox induction, indicating that short periods of Dox induction could potentially be used to identify primary target genes of transcription factors (Fig. 6C). Many tissues have poor permeability or delivery of Dox and are therefore exposed to lower Dox concentrations, thus strong activation of the Tet-On protein at low Dox concentrations is important for inducible expression in some tissues. Using the FLP-Inducer-NPAS4–3xFlag mES cells we found robust protein expression following 24hrs of treatment with Dox at concentrations as low as 10ng/ml (Fig. 6D).

## Discussion

Here we have engineered optimised, broadly applicable platforms for generation of Dox-inducible expression in immortalised cell lines, primary neurons and use with RMCE at a specific site in embryonic stem cells. Using a 3^rd^ generation Tet-activator system we have created lentiviral constructs capable of generating near homogenous Dox-inducible cell lines free of background expression and capable of strong and reversible ectopic expression or gene knockdown. We have also engineered a flexible Flp-In RMCE system for generation of site-specific Dox-inducible ectopic expression or gene knockdown in mouse embryonic stem cells. While similar lentiviral platforms have been described previously [23,34,35], our platform utilises the Tet-On 3G variant [3] which is demonstrably more sensitive to Dox and has lower background expression than other Tet-On variants. We also used the optimised TRE3G Tet-responsive promoter, which exhibits lower background expression [5]. We demonstrate that in the absence of Dox, expression of cDNAs from our lentiviral platform in stable cell lines is largely indistinguishable from uninfected parent cell lines, demonstrating absence of background leaky expression that often plagues these systems. Importantly, we describe an shRNA prediction tool that can effectively predict high potency shRNA target sequences when imbedded in the miR30 context, and we show that more than half of the sequences tested had the ability to knockdown gene expression from single copy, Dox-inducible cassette in embryonic stem cells. This prediction tool may significantly reduce the number of shRNA sequences needed to be tested to identify potent miR30c shRNA sequences for use *in vitro* and *in vivo*. In addition, application of this prediction tool to genome wide knockdown experiments may improve the identification of positive hits in screening experiments. Recently, through a series of optimisation experiments, miR30c based shRNA backbone and loop sequences have been further modified to improve processing and target gene knockdown independent of siRNA target sequence [36]. While we have not yet examined the effect of these modifications with our shRNA selection algorithm, we anticipate that this may further improve the efficiency of miR30 shRNA mediated gene knockdown.

Vector expression systems that utilise viral delivery are prone to stochastic gene silencing through epigenetic mechanisms, proviral integration-specific promoter interference, or genomic deletion due to selective pressure or genomic instability. These mechanisms result in heterogeneous expression in cell populations and have significantly hindered the use of lentiviral-based systems in studying genes essential for cell survival or proliferation. Here we have developed a lentiviral platform which overcomes many of these challenges by using a single vector expression system that contains all the components necessary for Dox-inducible expression, linked to either fluorescent or antibiotic-selectable markers, and absent of any background leaky expression. This allows for uniform Dox-inducible cell populations to be selected, which facilitates the investigation of LoF and GoF of genes essential for cell differentiation, proliferation or survival.

The FLP-Inducer system described here also provides a flexible platform, which allows for rapid and reversible Dox-inducible expression from a single defined locus. RMCE delivery of these constructs to the *Col1a1* locus in mES cells at efficiencies approaching 100% allows for facile generation of Dox-inducible cell lines. One of the key advantages of this system is that it can be readily adapted for use in targeting alternative genomic loci, which may be advantageous in certain tissues, or in creating multi-allelic Dox-inducible mice or generating site-specific FLP-In cell lines using FRT/FLP-mediated recombination. Expression of the Tet-activator and Tet-responsive factor(s) from a single locus simplifies the system and reduces potential positional effects, which may lead to silencing of transgene expression. The use of this high sensitivity, low background Tet-On 3G system also allows for reduced Doxycycline levels to be used *in vivo* or *in vitro* to gain strong Dox-inducible expression, minimising potential Doxycycline off-target effects and/or toxicity. Furthermore, the activity of the Tet-On 3G should be sufficient to gain Dox-inducible expression even with limited Doxycycline delivery in brain and some other peripheral tissues [2,22].

We have engineered our constructs to be Gateway-compatible, allowing the use of existing publicly available Gateway-compatible mouse and human ORF libraries (http://www.orfeomecollaboration.org) [37,38]. This broadens the potential use of this system, as it requires minimal molecular biology experience to generate GoF or LoF constructs. Importantly, the current promoters in these RMCE constructs can easily be exchanged for tissue- or cell type-specific promoters. Alternatively, the use of strong constitutive promoters (CAG and EF1α) followed by LoxP-STOP-LoxP (LSL) can be used in conjunction with tissue-specific CRE driver mice to gain tissue- or cell type-specific expression. There are now large numbers of CRE, tamoxifen inducible CRE Knock-In, or BAC-CRE driver lines publically available to gain specific tissue, regional or cell type specific expression (http://www.neuroscienceblueprint.nih.gov; http://www.gensat.org/cre.jsp; http://cre.jax.org; http://www.creportal.org/ [39–41]). We believe this added flexibility may address some of the problems encountered with previous Tet-On systems and broaden the potential application of this technology. The integration of low background Tet responsive promoters and the sensitive Tet-On 3G into all-in-one plasmids to deliver constructs by lentiviral transduction or RMCE significantly improves on existing systems [2,34,42]. The emergence of technologies allowing for efficient site specific pronuclear transgene delivery by integrase [43], CRISPR [44] or TALEN [45] technologies may also expedite the generation of Dox-inducible animal models and inbred mouse strains commonly used in immunology and neuroscience research. These systems advance those used in mouse genetics to allow complex biological questions to be addressed *in vivo* in a similar fashion to that of less complex model organisms.

## Supporting Information

S1 Fig Generation of multiple SIM2 Dox-inducible breast and prostate cancer cell lines using LVTPTIP lentivectors. (a,b,c) SIM2s-3xFlag or SIM2l-3xFlag containing LVTPTIP lentiviruses were infected into (a) prostate DU145 or (b) breast MDA-MB-231, MCF7, MDA-MB-453 cancer cell lines and selected with Puromycin. SIM2 expression was induced with 1μg/ml Dox (a) and (b) or 100ng/ml Dox (c) for indicated time. SIM2s, SIM2l (α-Flag) and tubulin protein levels were detected using western blot. In (b) short (upper panel) or long (lower panel) exposure times were used to assess background expression.
(TIF)

S2 Fig Schematic of Dox-inducible lentivectors and FLP-Inducer vectors. All FLP-Inducer plasmids were constructed with a 5’ PGK promoter + ATG and single FRT recombination site followed two SV40 PolyA sequences, the TRE3G promoter which has minimised background expression[5], Gateway recombination cassette (ccdB death gene and Chloramphenicol (Cm) antibiotic flanked byAttR1 and AttR2 LR recombination sites) and a SV40 PolyA sequence. 3’ of this lay various promoters which drive the expression of the Tet-On 3G which is followed by an SV40 PolyA sequence. FLP-Inducer-EF-LSL contains a constitutive EF1α promoter followed by a LoxP flanked SV40 PolyA sequence; FLP-Inducer-CAG and FLP-Inducer-CAG-LSL contain a cytomegalovirus (CMV) intermediate enhancer chicken β-Actin promoter (FLP-Inducer-CAG-LSL also contains a LoxP flanked SV40 PolyA sequence following the CAG promoter); FLP-Inducer-hGFAP contains a human Glial fibrillary acidic protein (GFAP) promoter; FLP-Inducer-hSynI contains a human Synapsin I promoter and FLP-Inducer-ARR2Pb contains a minimal rat Probasin promoter (Pb) with two Androgen Receptor response element enhancers (ARR2)[46].
(TIF)

S3 Fig Inducible and reversible expression of dsRed in Col1a1-FLP-Inducer embryonic stem cells. FACS analysis of dsRed expression in Col1a1-FLP-Inducer-EF (dsRed-shGL3) embryonic stem cells in (a) “On” 1μg/ml Doxycycline for 0 (filled red), 4 (blue), 8 (orange), 24 (light green), 48 (dark green) hrs; (b) “On” 1μg/ml Doxycycline for 0 (blue), 48 (filled light green) hrs and “Off” Doxycycline for 24 (red) or 48 (orange) hrs; (c) “On” 1μg/ml Doxycycline for 0 (blue) or 6 (orange) days and “Off” Doxycycline for 6 (red) days. (d) Median dsRed fluorescence in Col1a1-FLP-Inducer-EF(dsRed-shGL3) embryonic stem cells “On” 1μg/ml Doxycycline for 0 or 6 days and “Off” Doxycycline for 6 days. Data are average median dsRed fluorescence ± SD of 3 independent experiments in (d).
(TIF)

S4 Fig Cre mediated excision of LoxP-STOP-LoxP enables inducible expression in embryonic stem cells. (a) Schematic diagram of CRE mediated excision of a LoxP flanked STOP sequence which then enables Dox-inducible expression. (b) Bright field (left) or fluorescence (right) of mES-Col1a1-FLP-Inducer-LSL-dsRed-shP53 (814) cells treated with 1μg/ml Dox for 24hrs (bottom 2 panels) or left untreated (top). The bottom panel cells have been electroporated with a CRE expressing plasmid prior to treatment with Dox. (c) RT-PCR of genomic DNA extracted from mES-Col1a1-FLP-Inducer-LSL-dsRed-shP53 cells electroporated (+) or not electroporated (-) with a CRE expressing plasmid.
(TIF)

S1 Table shRNA oligonucleotides. (PDF)
