# Supplementary figures and images for: Inducible and Reversible Lentiviral and Recombination Mediated Cassette Exchange (RMCE) Systems for Controlling Gene Expression
Source: PLoS One. 2015 Mar 13;10(3):e0116373. doi: 10.1371/journal.pone.0116373 (PMC4358958; doi:10.1371/journal.pone.0116373)

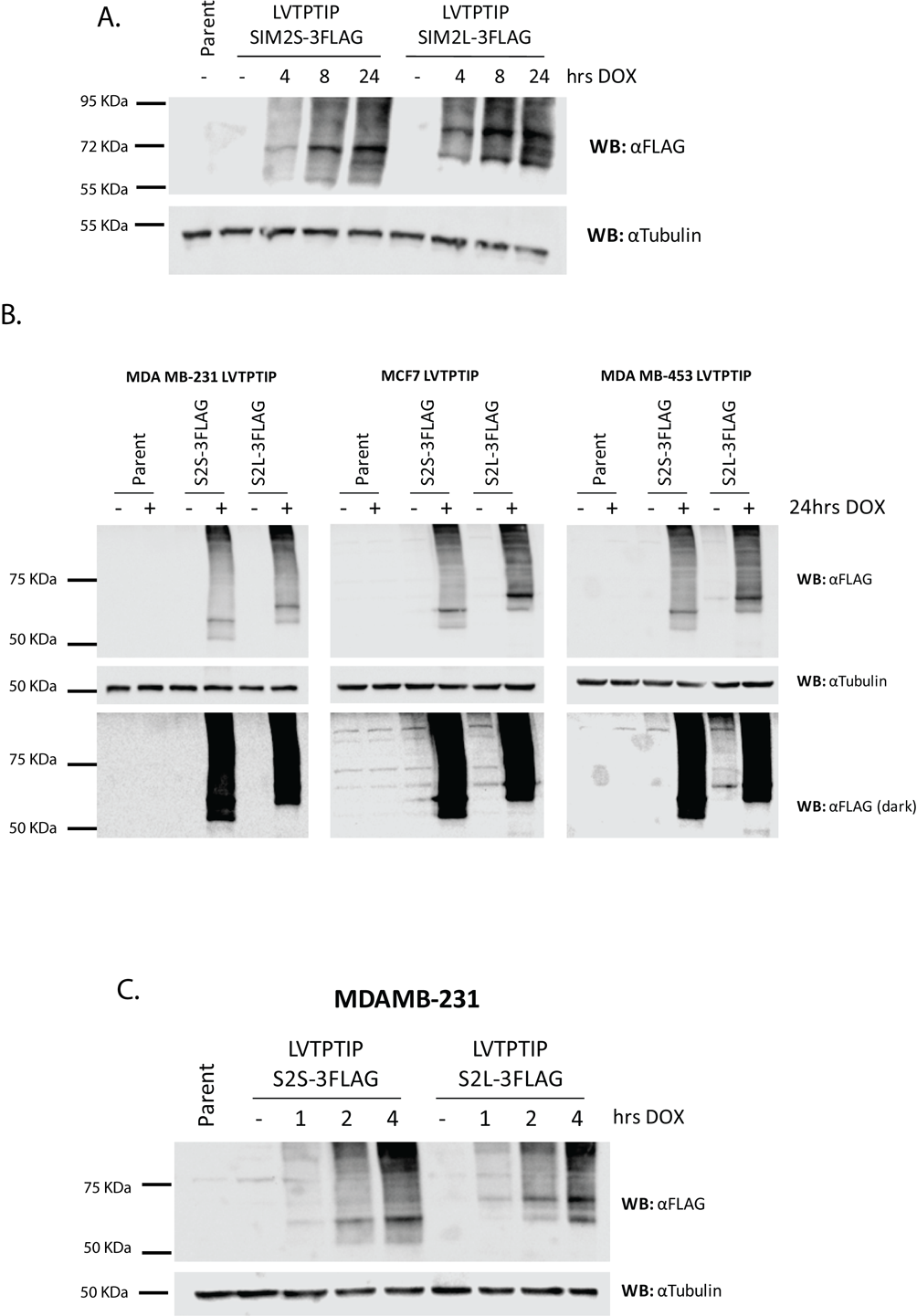

Supplement: S1 Fig — (a,b,c) SIM2s-3xFlag or SIM2l-3xFlag containing LVTPTIP lentiviruses were infected into (a) prostate DU145 or (b) breast MDA-MB-231, MCF7, MDA-MB-453 cancer cell lines and selected with Puromycin. SIM2 expression was induced with 1μg/ml Dox (a) and (b) or 100ng/ml Dox (c) for indicated time. SIM2s, SIM2l (α-Flag) and tubulin protein levels were detected using western blot. In (b) short (upper panel) or long (lower panel) exposure times were used to assess background expression. (TIF) [file pone.0116373.s001.tif]

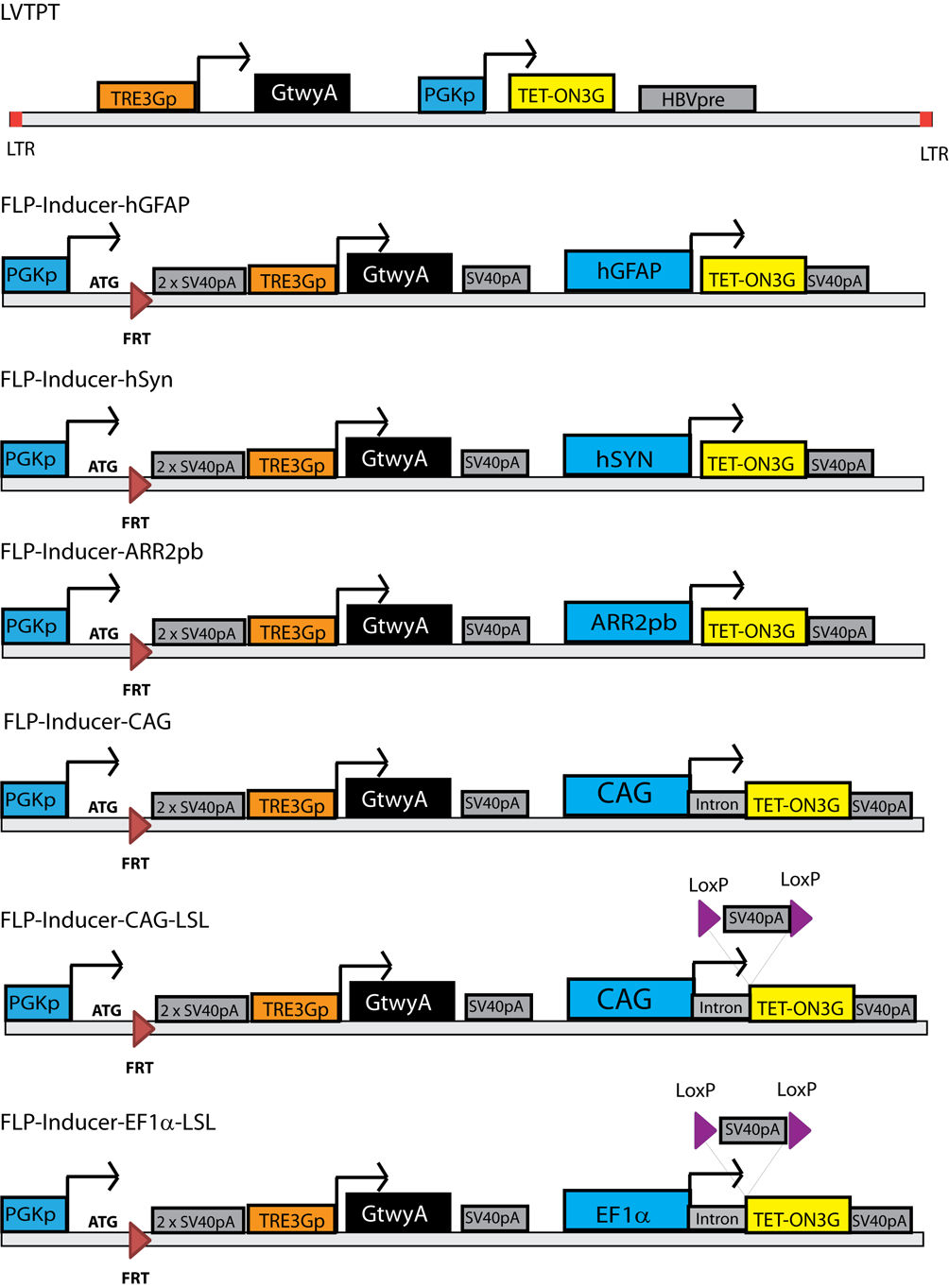

Supplement: S2 Fig — All FLP-Inducer plasmids were constructed with a 5’ PGK promoter + ATG and single FRT recombination site followed two SV40 PolyA sequences, the TRE3G promoter which has minimised background expression[5], Gateway recombination cassette (ccdB death gene and Chloramphenicol (Cm) antibiotic flanked byAttR1 and AttR2 LR recombination sites) and a SV40 PolyA sequence. 3’ of this lay various promoters which drive the expression of the Tet-On 3G which is followed by an SV40 PolyA sequence. FLP-Inducer-EF-LSL contains a constitutive EF1α promoter followed by a LoxP flanked SV40 PolyA sequence; FLP-Inducer-CAG and FLP-Inducer-CAG-LSL contain a cytomegalovirus (CMV) intermediate enhancer chicken β-Actin promoter (FLP-Inducer-CAG-LSL also contains a LoxP flanked SV40 PolyA sequence following the CAG promoter); FLP-Inducer-hGFAP contains a human Glial fibrillary acidic protein (GFAP) promoter; FLP-Inducer-hSynI contains a human Synapsin I promoter and FLP-Inducer-ARR2Pb contains a minimal rat Probasin promoter (Pb) with two Androgen Receptor response element enhancers (ARR2)[46]. (TIF) [file pone.0116373.s002.tif]

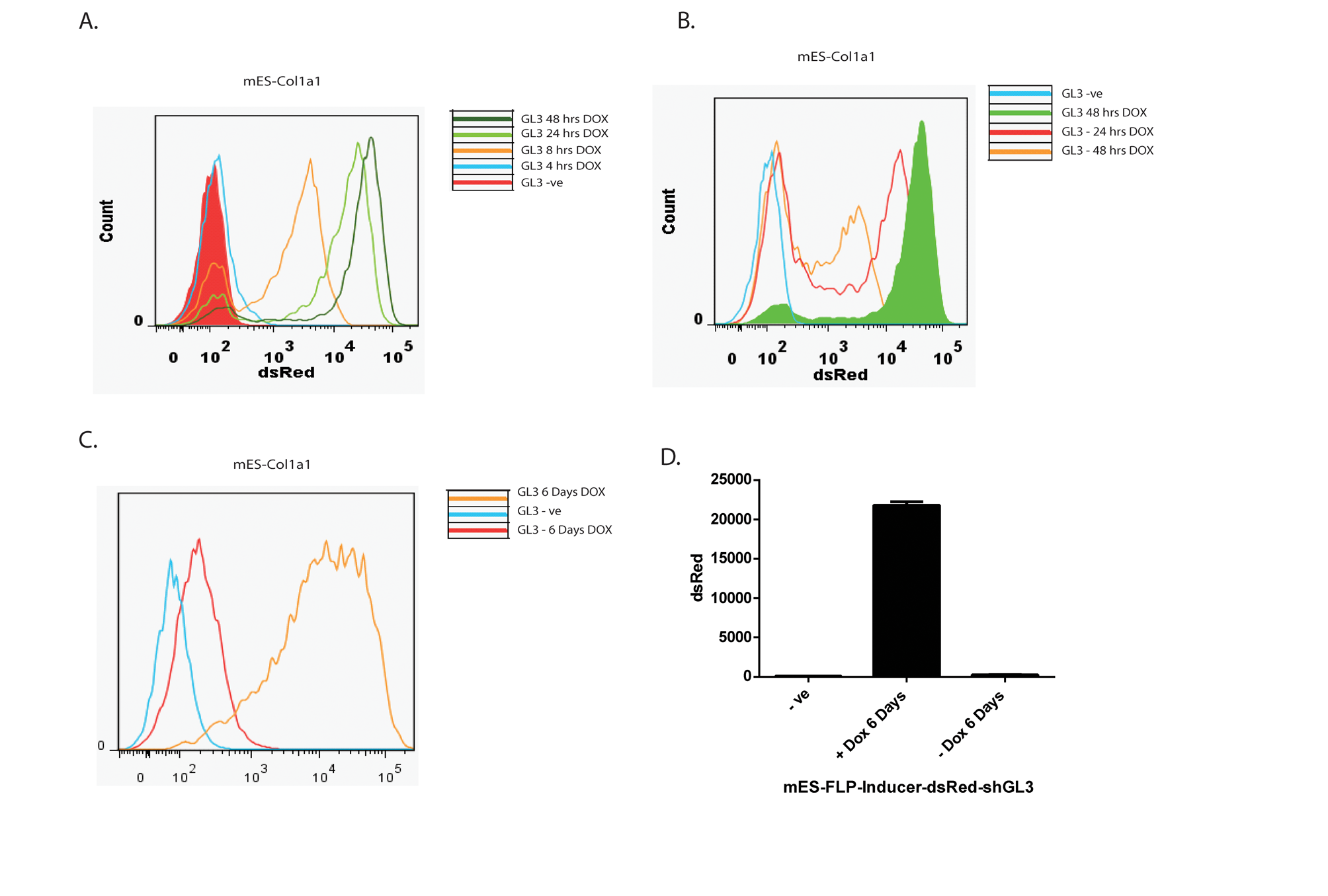

Supplement: S3 Fig — FACS analysis of dsRed expression in Col1a1-FLP-Inducer-EF (dsRed-shGL3) embryonic stem cells in (a) “On” 1μg/ml Doxycycline for 0 (filled red), 4 (blue), 8 (orange), 24 (light green), 48 (dark green) hrs; (b) “On” 1μg/ml Doxycycline for 0 (blue), 48 (filled light green) hrs and “Off” Doxycycline for 24 (red) or 48 (orange) hrs; (c) “On” 1μg/ml Doxycycline for 0 (blue) or 6 (orange) days and “Off” Doxycycline for 6 (red) days. (d) Median dsRed fluorescence in Col1a1-FLP-Inducer-EF(dsRed-shGL3) embryonic stem cells “On” 1μg/ml Doxycycline for 0 or 6 days and “Off” Doxycycline for 6 days. Data are average median dsRed fluorescence ± SD of 3 independent experiments in (d). (TIF) [file pone.0116373.s003.tif]

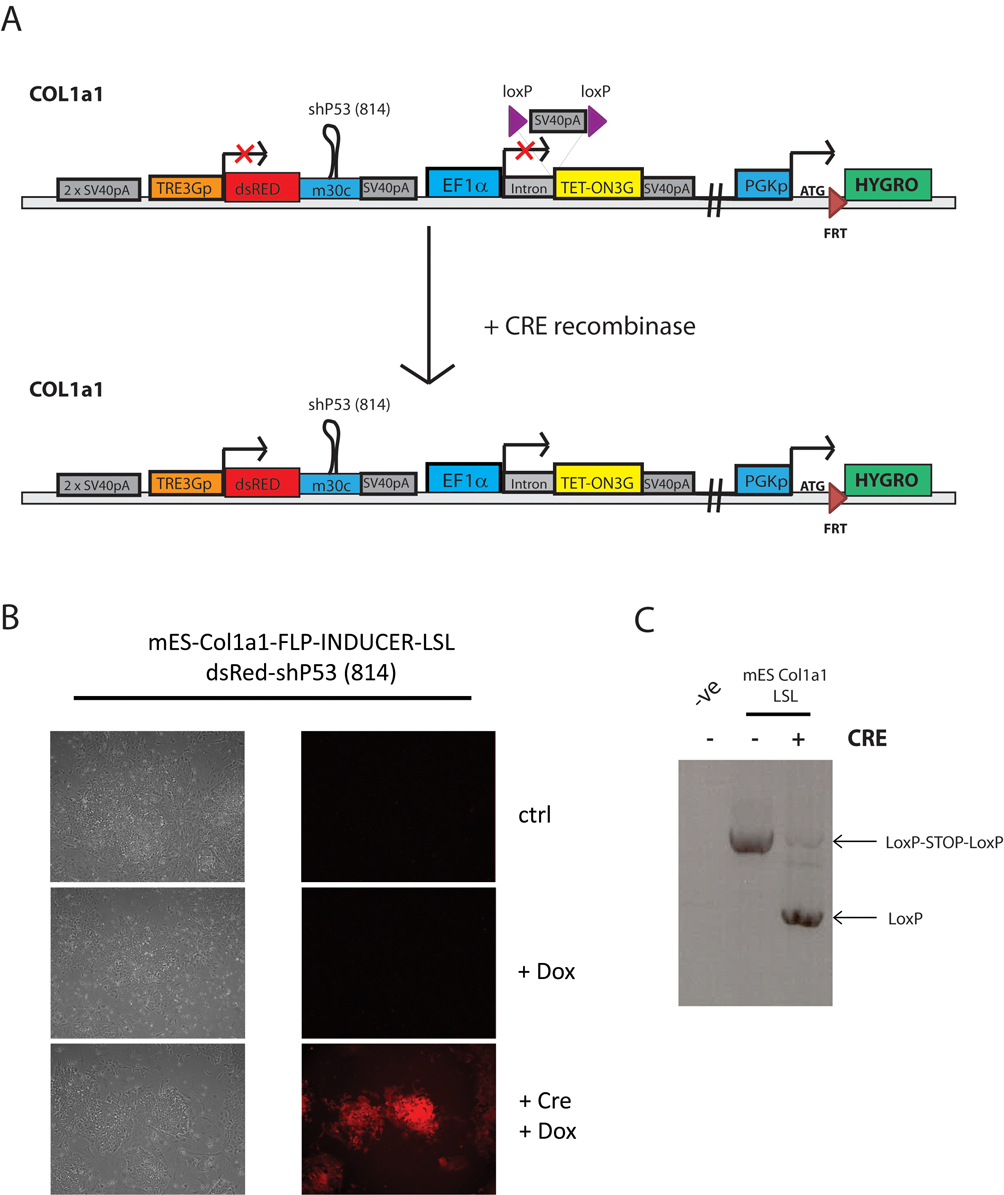

Supplement: S4 Fig — (a) Schematic diagram of CRE mediated excision of a LoxP flanked STOP sequence which then enables Dox-inducible expression. (b) Bright field (left) or fluorescence (right) of mES-Col1a1-FLP-Inducer-LSL-dsRed-shP53 (814) cells treated with 1μg/ml Dox for 24hrs (bottom 2 panels) or left untreated (top). The bottom panel cells have been electroporated with a CRE expressing plasmid prior to treatment with Dox. (c) RT-PCR of genomic DNA extracted from mES-Col1a1-FLP-Inducer-LSL-dsRed-shP53 cells electroporated (+) or not electroporated (-) with a CRE expressing plasmid. (TIF) [file pone.0116373.s004.tif]
