# Supplementary material for: Inducible and Reversible Lentiviral and Recombination Mediated Cassette Exchange (RMCE) Systems for Controlling Gene Expression
Source: PLoS One. 2015 Mar 13;10(3):e0116373. doi: 10.1371/journal.pone.0116373 (PMC4358958; doi:10.1371/journal.pone.0116373)
Supplement: S1 Table — (PDF) [file pone.0116373.s005.pdf]

miR30 shRNA template	Sequence
shGL3 (1309)	TGCTGTTGACAGTGAGCGCCCGCCTGAAGTCTCTGATTAATAGTGAAGCCACAGATGTATTAATCAGAGACTTCAGGCGGTTGCCTACTGCCTCGGA
P53 shRNA 814	TGCTGTTGACAGTGAGCGCCCACTACAAGTACATGTGTAATAGTGAAGCCACAGATGTATTACACATGTACTTGTAGTGGATGCCTACTGCCTCGGA
NPAS4 shRNA 275134	TGCTGTTGACAGTGAGCGCCGCCGAGATTCGGAACCTCAATAGTGAAGCCACAGATGTATTGAGGTTCCGAATCTCGGCGTTGCCTACTGCCTCGGA
NPAS4 shRNA 2182	TGCTGTTGACAGTGAGCGCGGTTGACCCTGATAATTTATTTAGTGAAGCCACAGATGTAAATAAATTATCAGGGTCAACCATGCCTACTGCCTCGGA
mARNT1 (4033)	TGCTGTTGACAGTGAGCGCGGAGGCTATTTTTTTATTCTATAGTGAAGCCACAGATGTATAGAATAAAAAAATAGCCTCCTTGCCTACTGCCTCGGA
mARNT1 (3284)	TGCTGTTGACAGTGAGCGCCCCAGCCAAAATGGAACTTTATAGTGAAGCCACAGATGTATAAAGTTCCATTTTGGCTGGGTTGCCTACTGCCTCGGA
mARNT1 (3751)	TGCTGTTGACAGTGAGCGCCAGGGTCACTTGATAATTATATAGTGAAGCCACAGATGTATATAATTATCAAGTGACCCTGTTGCCTACTGCCTCGGA
mARNT1 (415)	TGCTGTTGACAGTGAGCGACCAGGGAAAATCATAGTGAAATAGTGAAGCCACAGATGTATTTCACTATGATTTTCCCTGGCTGCCTACTGCCTCGGA
mARNT1 (282)	TGCTGTTGACAGTGAGCGCGGGCTGGATTTTGATGATGAATAGTGAAGCCACAGATGTATTCATCATCAAAATCCAGCCCTTGCCTACTGCCTCGGA
mARNT1 (414)	TGCTGTTGACAGTGAGCGCGCCAGGGAAAATCATAGTGAATAGTGAAGCCACAGATGTATTCACTATGATTTTCCCTGGCATGCCTACTGCCTCGGA
mARNT1 (522)	TGCTGTTGACAGTGAGCGCCCAGACAAGCTAACCATCTTATAGTGAAGCCACAGATGTATAAGATGGTTAGCTTGTCTGGTTGCCTACTGCCTCGGA
mARNT1 (1498)	TGCTGTTGACAGTGAGCGCCAGATGAAATTGAGTATATTATAGTGAAGCCACAGATGTATAATATACTCAATTTCATCTGATGCCTACTGCCTCGGA
mARNT1 (3235)	TGCTGTTGACAGTGAGCGCTGCAGCCAAAGAGTATTTAAATAGTGAAGCCACAGATGTATTTAAATACTCTTTGGCTGCAATGCCTACTGCCTCGGA
mARNT1 (300)	TGCTGTTGACAGTGAGCGCGAAGTAGAAGTGAACACTAAATAGTGAAGCCACAGATGTATTTAGTGTTCACTTCTACTTCATGCCTACTGCCTCGGA
mARNT2 (2221)	TGCTGTTGACAGTGAGCGCCGCAGGGGACTGGCAACTATATAGTGAAGCCACAGATGTATATAGTTGCCAGTCCCCTGCGTTGCCTACTGCCTCGGA
mARNT2 (5565)	TGCTGTTGACAGTGAGCGCGGGCAACATAATGAAACTAATTAGTGAAGCCACAGATGTAATTAGTTTCATTATGTTGCCCATGCCTACTGCCTCGGA
mARNT2 (377)	TGCTGTTGACAGTGAGCGAGCGGAACAAGATGACTCAATATAGTGAAGCCACAGATGTATATTGAGTCATCTTGTTCCGCCTGCCTACTGCCTCGGA
mARNT2 (4696)	TGCTGTTGACAGTGAGCGCCAATGCCATTATCCTGCAAAATAGTGAAGCCACAGATGTATTTTGCAGGATAATGGCATTGATGCCTACTGCCTCGGA
mARNT2 (4612)	TGCTGTTGACAGTGAGCGAGGGGGAGAAGTCAGAAATGAATAGTGAAGCCACAGATGTATTCATTTCTGACTTCTCCCCCTGCCTACTGCCTCGGA
mARNT2 (4329)	TGCTGTTGACAGTGAGCGCGGACAGGATTCTTGCTTGCTATAGTGAAGCCACAGATGTATAGCAAGCAAGAATCCTGTCCTTGCCTACTGCCTCGGA
mARNT2 (3708)	TGCTGTTGACAGTGAGCGCCCCTGTGTCAGTCAGATTTAATAGTGAAGCCACAGATGTATTAAATCTGACTGACACAGGGATGCCTACTGCCTCGGA
mARNT2 (3186)	TGCTGTTGACAGTGAGCGAGGTTAGGATACCTCGACTCTATAGTGAAGCCACAGATGTATAGAGTCGAGGTATCCTAACCCTGCCTACTGCCTCGGA
mARNT2 (318)	TGCTGTTGACAGTGAGCGCGATGGTGAAGGTCCCAGTAAATAGTGAAGCCACAGATGTATTTACTGGGACCTTCACCATCTTGCCTACTGCCTCGGA
mARNT2 (4611)	TGCTGTTGACAGTGAGCGCGGGGGGAGAAGTCAGAAATGATAGTGAAGCCACAGATGTATCATTTCTGACTTCTCCCCCCATGCCTACTGCCTCGGA
